# Shoulder Septic Arthritis in a Child: A Diagnostic Dilemma

**DOI:** 10.7759/cureus.42986

**Published:** 2023-08-05

**Authors:** Ahmad Azraf Azhar, Kamal Jamil, Ahmad Fazly Abd Rasid

**Affiliations:** 1 Orthopaedics and Traumatology, Pusat Perubatan Universiti Kebangsaan Malaysia, Kuala Lumpur, MYS; 2 Paediatric Orthopaedics, Pusat Perubatan Universiti Kebangsaan Malaysia, Kuala Lumpur, MYS

**Keywords:** osteomyelitis, arthrotomy, shoulder pseudo paralysis, children joint infections, shoulder septic arthritis

## Abstract

Septic arthritis of the shoulder in pediatric patients should be diagnosed and treated urgently to prevent complications of the disease. However, early detection can be a challenge due to mild symptoms with ambiguous laboratory and radiological findings. We report a case of an eight-month-old girl who presented to us initially with pseudo paresis of her right shoulder without any signs suggestive of infection. After a negative ultrasound, she was discharged with analgesia upon improvement of range of motion. Three weeks later, she presented with recurrent shoulder pain associated with fever, swelling, elevated CRP, and osteomyelitis changes of the humeral head on a plain radiograph. We proceeded with a minimally invasive arthrotomy washout and commenced on IV antibiotics. At one month follow-up, she regained her full range of motion and recovered fully. No recurrence of septic arthritis until six-month follow-up. This write-up discusses the diagnostic challenge of pediatric shoulder septic arthritis and the surgical technique of minimally invasive arthrotomy washout in a pediatric patient.

## Introduction

Septic arthritis in children is an emergency that requires early diagnosis and prompt treatment due to high rates of local and systemic complications. In cases of shoulder septic arthritis, delayed treatment can lead to osteomyelitis, physeal arrest, and subluxation of the affected shoulder [1]. However, diagnosing septic arthritis in this age group is a challenge because presentations are usually subtle with relatively insensitive laboratory and imaging studies. There is also a paucity of literature on antibiotics treatment duration and surgical approach, particularly in shoulder septic arthritis. Thus, this report aims to share our experience in managing the case of an eight-month-old girl.

## Case presentation

**Figure 1:**
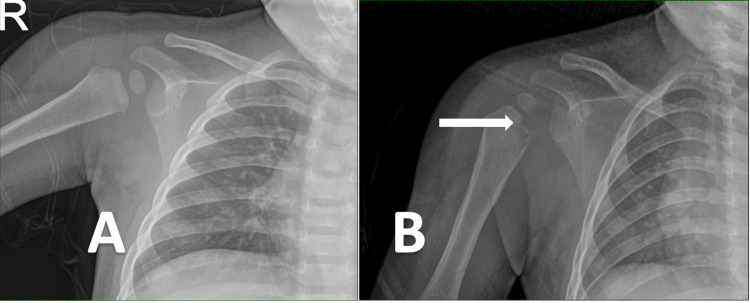
Plain anteroposterior radiograph of the right shoulder at first presentation (A) and second presentation (B). In radiograph B, there was a lytic lesion over the proximal humerus indicated by the arrow.

An eight-month-old girl was brought to the emergency department by her parents with a two-day history of a limited range of motion of the right shoulder. The parents noticed the symptom after picking up the patient from the daycare center. They denied any prior trauma, and the patient has no history of recent infection such as viral fever or upper respiratory tract infection. Clinically, no swelling and erythema over the affected shoulder but it was warm when compared to the contralateral side. Generalized tenderness and limited active movement were noted over the affected shoulder. Laboratory investigations showed a white blood cell count of 15.3×10^9^/L, mildly elevated CRP value of 1.53 mg/dL (normal value: <0.5mg/dL), and erythrocyte sedimentation rate (ESR) of 78 mm/hr (Normal value: <10 mm/hr). The shoulder radiograph did not show any signs of joint infection or osteomyelitis (Figure 1A). A contralateral shoulder radiograph was also done for comparison and both appeared normal. She was admitted to the ward for further observation and investigations. During admission, an ultrasound of the right shoulder was done and intramuscular edema at the subscapularis region was reported, which is suggestive of soft tissue injury. She received analgesia but was not started on any antibiotic and was subsequently discharged home with a one-week follow-up appointment after her symptoms improved.

After three weeks, she presented to the emergency department again with recurrent pseudo paralysis but now associated with fever and right arm swelling. On examination, the right shoulder was warm, swollen, and erythematous. There was also a palpable swelling of approximately 3×2 cm over the medial arm region. CRP and ESR values were increased (4.89 mg/dL and 119 mm/hr, respectively) but white blood cell count was normal (9.3×10^9^/L). The shoulder radiograph revealed a metaphyseal lucency over the proximal humerus that could suggest osteomyelitis (Figure 1B) and repeated ultrasound showed a widening of joint space with intraarticular debris. Diagnosis of septic arthritis of the right shoulder was made but tuberculosis (TB) of the joint still needs to be ruled out. She was subsequently started on IV cefuroxime and underwent a minimally invasive joint washout.

**Figure 2:**
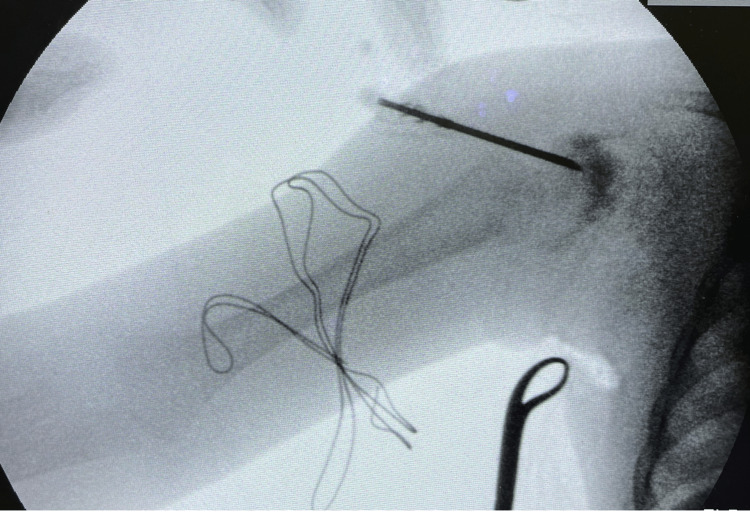
Location of the needle confirmed by radiopaque dye injection.

**Figure 3:**
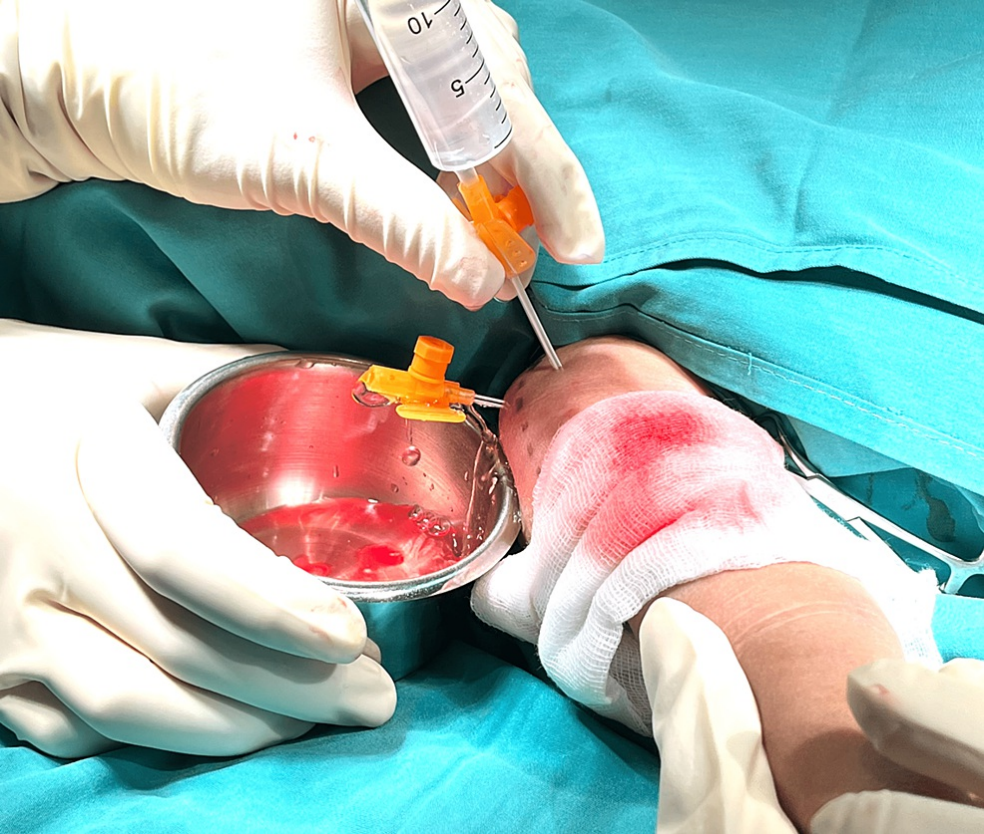
Minimally invasive shoulder joint washout.

The surgery was done as described by Yassa et al. [2]. The patient was placed in the supine position and put under general anesthesia. Two wide-bore cannulas were inserted into the anterior shoulder joint 3 cm apart. Joint fluid was aspirated and sent for microbiologic studies. Intraoperatively, we managed to aspirate 5 ml of pus discharge from the joint. The position of these needles was confirmed by radiopaque dye injection (Figure 2). The trocar was then removed leaving the plastic sheath inside the joint. One portal was used for washout and the other for drainage and aspiration (Figure 3). A joint washout was performed thoroughly with simultaneous manipulation of the joint to ensure a complete washout. Last, suction is attached to the outgoing cannula to remove all remaining fluid.

**Figure 4:**
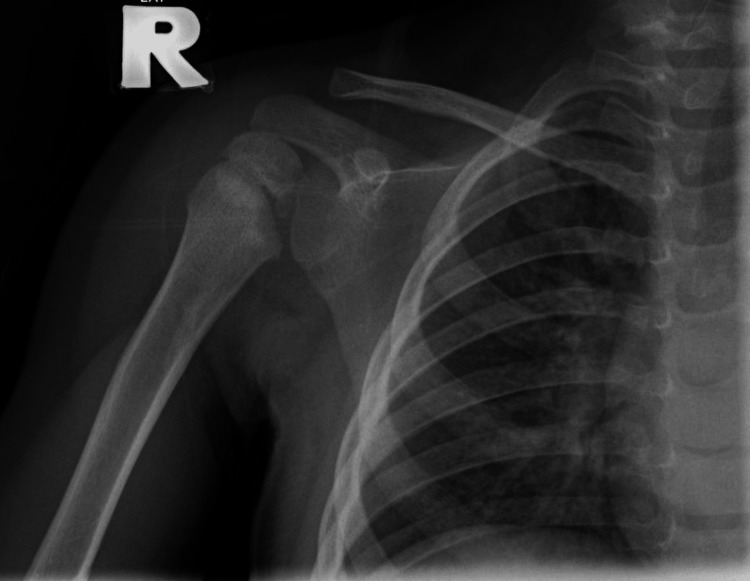
Right shoulder radiograph at one-year follow-up.

Post-operatively, she received eight days of IV cefuroxime and her CRP value dropped to 0.07 mg/dL upon discharge. No organism was isolated from her culture samples. TB PCR also came back negative. She was afebrile and had a full range of motion before discharge. She was prescribed oral antibiotics for a total duration of six weeks. At a one-year follow-up, the patient's wound has healed, and she has a full range of motion in the right shoulder. The repeated right shoulder radiograph showed that the metaphyseal lucency is reducing and epiphysis was intact (Figure 4).

## Discussion

Septic arthritis of the shoulder in pediatric patients can be difficult to diagnose because they usually presented with mild symptoms and overlapping infections. There are a few parameters that are predictive of septic arthritis such as pseudo paralysis with fever, elevated CRP value, and osteomyelitis changes on ultrasound or radiograph [3]. However, the most predictive parameters are still unclear, and there is limited literature available to establish a diagnostic algorithm.

Based on the retrospective case series by Danilov et al., the most predictive indicators are pseudo paralysis and elevated CRP value [4]. All 25 patients in the study presented with a limited range of motion, and 24 cases has elevated CRP value. In another study, Levine et al. suggested that CRP is a better independent predictor of the disease than ESR [5]. Increased leucocyte count would be supportive but otherwise a poor independent positive predictor [4]. Other than that, symptoms such as fever might be predictive of a joint infection, but nonspecific. Children may present without fever due to an immature immune response to infection [6].

In this case report, the patient presented with pseudo paralysis but did not have a notably high CRP value or radiographic changes indicative of septic arthritis during her first presentation. After a negative ultrasound and a good recovery, she was eventually discharged from the ward. We were only able to diagnose her with septic arthritis of the right shoulder on her second visit when she presented with swelling, high CRP, and osteomyelitis changes in the shoulder radiograph. This scenario clearly emphasizes the challenges in diagnosing shoulder septic arthritis and the importance of maintaining a high index of suspicion in pediatric patients.

Imaging such as a plain radiograph, ultrasound, and magnetic resonance imaging (MRI) of the affected joint can all provide information to assist the diagnosis. However, on a plain radiograph, infective or osteomyelitis changes do not usually present in the early stage of the infection. Belthur et al. discovered that septic arthritis of the glenohumeral joint needs a significantly longer time from the onset of symptoms to manifest on a radiograph [7]. There are studies done to compare the effectiveness of an ultrasound or MRI in detecting shoulder septic arthritis. Danilov et al. reported that ultrasound is almost as effective as an MRI in diagnosing septic arthritis of the shoulder [4]. In their studies, 15 out of 21 patients had positive ultrasound findings while all 15 patients who underwent MRI had features of septic arthritis [3].

Ernat et al. found that MRI is the most accurate modality in diagnosing osteoarticular infections [8]. Practically, ultrasound is readily available and can be done on the same day, but it is highly user dependent. In our patient, the ultrasound did not aid the diagnosis during the initial presentation. On the other hand, MRI is highly accurate, although it is not usually done on the same day. Hence, there will be delays in making diagnosis and initiating treatment for patients. In retrospect, an MRI might be useful to confirm the diagnosis in our case after the negative ultrasound results.

The main treatment of septic arthritis is adequate drainage and antibiotics. However, there is only limited study done on the best drainage or washout method for septic arthritis of the shoulder in children. Arthrotomy offers comprehensive joint visualization and debridement but it also comes with increased invasiveness and potential complications such as stiffness of the joint. On the other hand, minimally invasive washout is less invasive and can offer quicker recovery but some may argue that it may have limitations in treating complex infections. Bos et al. reported that all children who are treated with aspiration have a negligible loss of function during long-term follow-up [9]. Aspiration can be supplemented with lavage or washout through the needle to ensure adequate evacuation of bacteria and debris from the joint. Forward et al. also reported that arthroscopy washout can achieve adequate drainage and washout with minimal morbidity to the patient [10].

From this case, we can appreciate that diagnosing septic arthritis of the shoulder can be challenging especially when the signs of infection are not obvious. Thus, we might require further imaging such as ultrasound and MRI to help us to diagnose septic arthritis. In terms of treatment, a minimally invasive approach of joint washout can be effective and lead to a good short-term outcome for our patient.

## Conclusions

In summary, septic arthritis of the shoulder among children is a diagnostic challenge. Good predictive parameters such as limited movement of the joint and elevated CRP should point us toward septic arthritis. In cases of mild presenting symptoms and mildly raised infective markers, advanced imaging such as an ultrasound or an MRI is indicated. Diagnosis should be made effectively so that treatment can be started promptly to prevent further complications.
